# A Data‐Driven Threshold for Extreme Hyponatremia in the Emergency Department and Its Association With 28‐Day Mortality

**DOI:** 10.1002/kjm2.70291

**Published:** 2026-09-24

**Authors:** Yalcin Golcuk, Ömer‐Faruk Karakoyun, Fulden Cantaş‐Türkiş, İsmail Kırlı, Satuk‐Buğra Yıldırım, Bekir Baran, Burcu Kaymak‐Golcuk

**Affiliations:** ^1^ Department of Emergency Medicine, Faculty of Medicine Muğla Sıtkı Koçman University Muğla Türkiye; ^2^ Department of Health of the Elderly, Institute of Health Sciences Muğla Sıtkı Koçman University Muğla Türkiye; ^3^ Emergency Medicine Service Muğla Training and Research Hospital Muğla Türkiye; ^4^ Department of Biostatistics, Faculty of Medicine Muğla Sıtkı Koçman University Muğla Türkiye; ^5^ Department of Internal Medicine, Faculty of Medicine Muğla Sıtkı Koçman University Muğla Türkiye; ^6^ Clinical Biochemistry Service Muğla Training and Research Hospital Muğla Türkiye

**Keywords:** emergency department, extreme hyponatremia, hyponatremia, mortality, risk stratification

## Abstract

Hyponatremia is a common electrolyte disorder in the emergency department (ED), but its clinical burden is not evenly distributed across the sodium spectrum. We conducted a retrospective observational cohort study of adult ED encounters at a tertiary referral hospital between July 2019 and July 2024 to identify a data‐driven threshold for extreme hyponatremia and evaluate its association with 28‐day mortality. Encounters with simultaneous sodium and glucose measurements were included, and sodium values were corrected for hyperglycemia. Kernel density estimation identified a distinct local minimum defining distribution‐derived extreme hyponatremia (DDEH). Propensity score matching based on age and sex was used to compare DDEH with nonextreme hyponatremia and normonatremia. Among 227,027 eligible ED patients, density modeling identified an inflection point at 110 mmol/L. Sixty‐one patients (0.027%) met criteria for DDEH. In matched analyses (*n* = 61 per group), 28‐day mortality was highest in the DDEH group (36.1%), compared with nonextreme hyponatremia (18.0%) and normonatremia (6.6%) (*p* < 0.001). Kaplan–Meier analysis showed significantly reduced survival in patients with DDEH (log‐rank *p* < 0.001). A distribution‐derived sodium threshold of 110 mmol/L identified a rare but high‐risk phenotype of extreme hyponatremia in ED patients. This cohort‐derived boundary identified a rare extreme‐tail phenotype associated with systemic physiological vulnerability and increased short‐term mortality risk, but external validation is required before broader clinical implementation.

## 1 Introduction

Among electrolyte disorders encountered in the emergency department (ED), few combine high prevalence with prognostic significance as consistently as hyponatremia. Although common in ED populations, its clinical burden is not evenly distributed across the sodium spectrum and appears to concentrate at the lowest sodium levels, where physiological instability, diagnostic uncertainty, and mortality risk converge [1, 2, 3, 4]. Large population‐based investigations, including the Stockholm Sodium Cohort of approximately 1.6 million individuals, have enabled more robust estimation of outcomes across the dysnatremia spectrum [5]. These studies show that mortality increases nonlinearly as sodium declines, with adverse outcomes particularly pronounced in older adults and in patients with substantial comorbidity burden [6, 7, 8]. Hyponatremia is conventionally defined as serum sodium < 135 mmol/L and stratified as mild (130–135 mmol/L), moderate (125–129 mmol/L), and profound (< 125 mmol/L) [9]. More recent guideline updates have further designated severe hyponatremia as < 120 mmol/L [10]. Within this highest‐acuity segment, neurological deterioration, short‐term mortality, and treatment‐related complications such as osmotic demyelination syndrome associated with overly rapid correction may coexist, underscoring the need for more precise risk stratification in the ED [9, 10, 11].

A persistent challenge in emergency medicine is determining the point at which mortality risk begins to rise more sharply as sodium falls. Conventional threshold‐based classifications impose discrete categories on what is fundamentally a continuous biological risk gradient and may therefore obscure heterogeneity within the most extreme sodium values. ED studies commonly operationalize symptomatic hyponatremia as < 125 mmol/L [12], whereas more recent cohorts have used stricter thresholds, such as < 116 mmol/L, to enrich for patients with greater acuity and systemic illness burden [13]. At still lower concentrations, including ≤ 113 mmol/L in the most extreme subgroup, patients appear increasingly overrepresented among those with marked multisystem vulnerability [12]. These observations raise the possibility that the extreme tail of the sodium distribution represents a clinically distinct phenotype rather than merely the lower boundary of existing severity categories. In the ED, this uncertainty is amplified by nonspecific presentations, multimorbidity, and polypharmacy, all of which often require early stabilization and cautious sodium correction before definitive etiologic clarification is possible.

Accordingly, an important conceptual and operational gap remains: the absence of an empirically derived definition of extreme hyponatremia grounded in real‐world patient distributions and clinically meaningful outcome gradients. For emergency physicians making early triage, monitoring, and treatment decisions, identifying such a boundary may offer a practical marker of heightened physiological vulnerability beyond consensus‐based biochemical cutoffs alone.

To address this gap, we used kernel density estimation (KDE) to characterize the distribution of glucose‐corrected serum sodium values in a large ED cohort and to identify an empirically derived threshold corresponding to the extreme end of the hyponatremia spectrum. We then evaluated whether this distribution‐defined boundary identified a clinically distinct subgroup characterized by greater neurological impairment, increased systemic vulnerability, and higher short‐term mortality. The aim of this study was to identify a data‐driven threshold for extreme hyponatremia in ED patients and to evaluate its association with 28‐day mortality.

## 2 Methods

### 2.1 Study Design and Setting

This single‐center, retrospective observational cohort study was conducted in the ED of Muğla Training and Research Hospital, a university‐affiliated tertiary referral hospital in Muğla, Türkiye. The ED is a high‐volume, 50‐bed facility with an annual census of approximately 150,000 visits. The study period extended from July 1, 2019, to July 1, 2024.

The study protocol was approved by the Muğla Sıtkı Koçman University Faculty of Medicine Clinical Research Ethics Committee (Decision No: 240254/57) and conducted in accordance with the Declaration of Helsinki and its later amendments. Because of the retrospective design and use of de‐identified data, the requirement for written informed consent was waived. The study was reported in accordance with STROBE and RECORD recommendations [14, 15].

### 2.2 Selection of Participants

**FIGURE 1:**
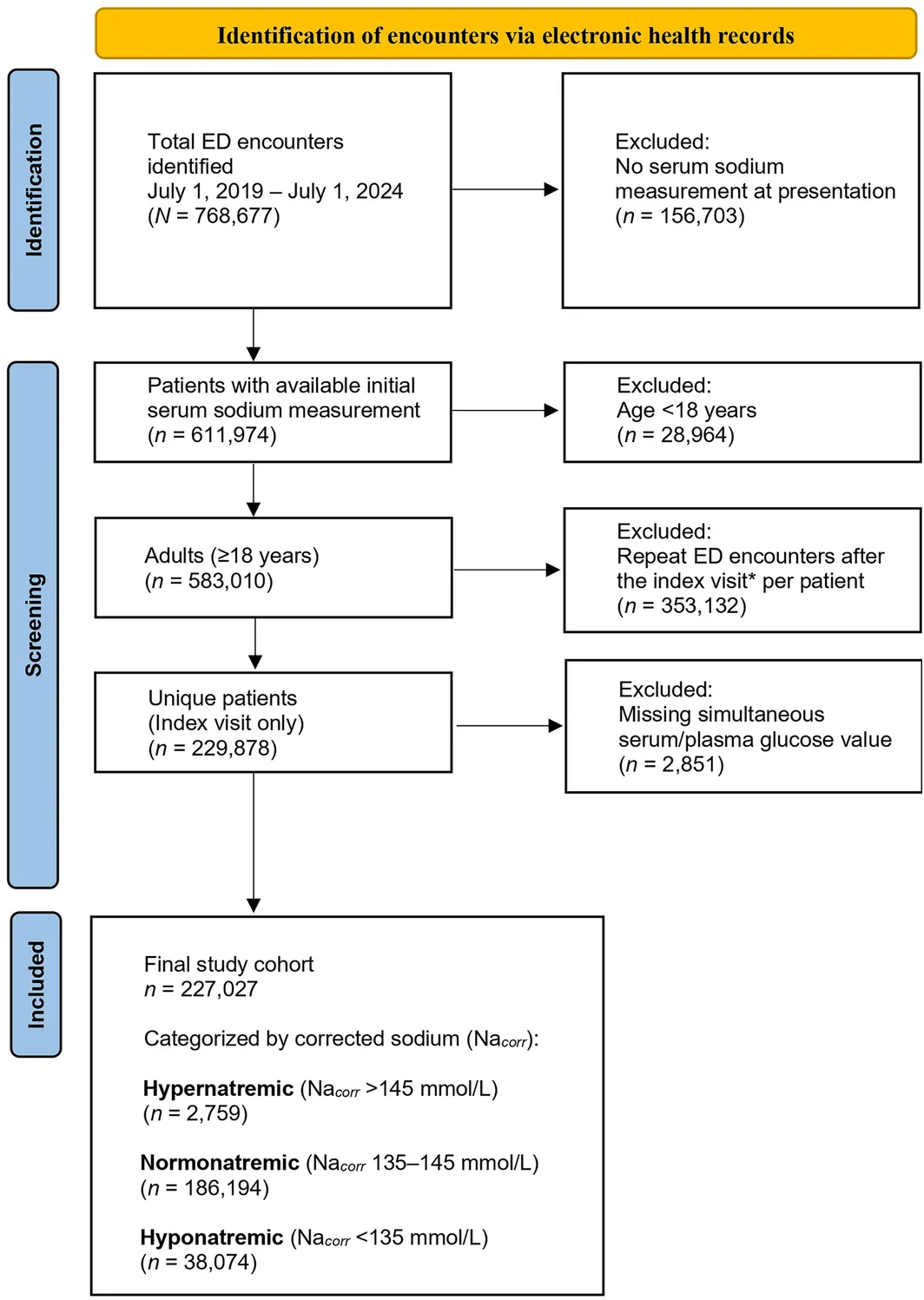
Cohort selection flow diagram. *Index visit was defined as the first eligible emergency department encounter for each unique patient during the study period; all subsequent visits by the same patient were excluded from the analytic cohort. ED, emergency department.

All adult ED encounters (age ≥ 18 years) during the study period were screened electronically through the hospital information system. Encounters were eligible if the index ED laboratory panel included both a serum sodium measurement and a contemporaneous plasma glucose value, allowing correction for hyperglycemia‐related translocational hyponatremia. To ensure independence of observations and reduce within‐patient clustering, only the first eligible ED encounter for each unique patient was included in the analytic cohort. This sampling framework corresponded to the cohort derivation shown in Figure 1.

### 2.3 Data‐Driven Threshold Derivation

To avoid reliance on predefined biochemical cutoffs, we used a distribution‐based approach to define sodium strata. Kernel density estimation (KDE) was applied to continuous glucose‐corrected sodium values to obtain a smoothed estimate of the cohort distribution [16, 17]. A Gaussian kernel was used, and bandwidth was selected according to Silverman's rule‐of‐thumb to standardize smoothing and preserve sensitivity to potential multimodality [18, 19]. Candidate thresholds were defined as local minima (antimodes) between adjacent density peaks. Because KDE‐derived cutoffs are distribution‐dependent, the resulting boundary was interpreted as cohort‐specific and was subsequently operationalized for comparative analyses [17, 18, 19, 20]. To evaluate the sensitivity of the KDE‐derived lower‐tail structure to bandwidth selection, additional KDE models were fitted using proportional bandwidth multipliers of 1.25, 1.50, and 2.00 relative to the original Silverman bandwidth. For each specification, local minima were re‐estimated from the smoothed density function. These sensitivity analyses were used to assess whether the lower‐tail separation of the corrected sodium distribution was preserved across alternative smoothing parameters.

### 2.4 Propensity Score Matching

To improve comparability across sodium strata, we performed propensity score matching (PSM) [21, 22]. The distribution‐defined extreme hyponatremia (DDEH) group served as the index cohort for two separate 1:1 matches against the nonextreme hyponatremia and normonatremia groups. Propensity scores were estimated by logistic regression using age and sex as covariates. Matching was performed by nearest‐neighbor matching without replacement on the logit of the propensity score [22, 23]. No caliper was applied in order to maximize retention in the small extreme‐tail subgroup, recognizing that caliper restriction may reduce sample size and destabilize matches in sparse datasets [24]. Balance after matching was assessed using standardized mean differences (SMDs), with values < 0.10 considered acceptable [22, 23]. In line with current methodological guidance, postmatching balance was prioritized over additional matching constraints [25]. Because the DDEH subgroup represented an extreme‐tail phenotype with limited sample size and event counts, the propensity score model was intentionally restricted to parsimonious demographic covariates in order to reduce instability and avoid overfitting. Inclusion of a larger number of covariates relative to the available event burden was considered likely to produce unstable matches, excessive pruning of observations, and reduced statistical efficiency in the extreme‐tail subgroup. Residual imbalance in illness‐severity variables was therefore interpreted cautiously, recognizing that demographic matching cannot fully eliminate confounding related to underlying systemic disease burden. Given residual between‐group differences in comorbidity burden and neurological status after matching, an additional parsimonious survival analysis was performed to evaluate whether the association between DDEH and 28‐day mortality persisted after limited covariate adjustment.

### 2.5 Data Collection and Clinical Variables

Eligible encounters were identified from the electronic medical record and abstracted into prespecified electronic case report forms. Two trained investigators independently abstracted the data while blinded to the study hypothesis and the empirically derived sodium threshold. The abstraction form was pilot‐tested in 20 randomly selected charts before full extraction. Disagreements were resolved by consensus with a third senior investigator, with final adjudication by a board‐certified emergency physician with more than 10 years of experience in fluid and electrolyte disorders. Interrater reliability was assessed in a randomly selected 10% subsample using Cohen's kappa for categorical variables and intraclass correlation coefficients for continuous variables. De‐identified data were stored on password‐protected institutional servers.

Abstracted variables included age, sex, Charlson Comorbidity Index (CCI), triage vital signs, Glasgow Coma Scale (GCS), and predefined symptoms potentially attributable to hyponatremia, including headache, lethargy, seizures, and loss of consciousness. Potential etiologic contributors were categorized using prespecified clinical criteria. However, because of the retrospective ED‐based design, detailed etiologic and pathophysiologic characterization variables, including chronicity of hyponatremia, volume status, urine sodium, urine osmolality, measured serum osmolality, medication chronology, endocrine testing, active malignancy, liver dysfunction, heart failure, and formal SIADH workup, were not consistently available across the full cohort. Therefore, these variables were not incorporated into the main comparative or multivariable modeling analyses. The primary outcome was 28‐day all‐cause mortality; the secondary outcome was hospital length of stay (LOS), defined as days from the index encounter to discharge or in‐hospital death.

Laboratory analyses were based on the first blood sample obtained at ED presentation. Variables were grouped as biochemical, hematologic, and calculated indices. Osmolality‐related parameters reported in this study were calculated from routinely available laboratory values; measured serum osmolality and urine osmolality were not routinely available in the ED records. Because hyperglycemia may produce translocational hyponatremia, glucose‐corrected sodium (Na_corr_) was used both for stratum definition and as the primary sodium exposure. Sodium correction was calculated using the Katz formula:

$${\mathrm{Na}}_{\text{corr}}=\text{Nameasured}+1.6\times \left(\left(\text{glucose}-100\right)/100\right)$$

where sodium is expressed in mmol/L and glucose in mg/dL.

### 2.6 Statistical Analysis

Statistical analyses were performed using R (v4.5.2; RStudio v2024.09.0, RStudio PBC, Boston, MA, USA) and IBM SPSS Statistics (v27.0; IBM Corp., Armonk, NY, USA). All sodium values were first corrected for serum glucose. KDE was then applied to the Na_corr_ distribution to derive empirical sodium thresholds. Bandwidth sensitivity analyses were subsequently performed using proportional bandwidth multipliers of 1.25, 1.50, and 2.00 to evaluate the stability of the lower‐tail density structure and the corresponding antimodal region. To reduce confounding by baseline demographics, PSM was subsequently performed in R using logistic regression and nearest‐neighbor matching without replacement via the MatchIt package.

Continuous variables were summarized as mean ± standard deviation or median (min–max), as appropriate after assessment of normality with the Shapiro–Wilk test and distributional diagnostics. Categorical variables were presented as counts and percentages. Group comparisons for continuous variables were performed using one‐way ANOVA or the Kruskal–Wallis test, as appropriate. Variance homogeneity was assessed with Levene's test; post hoc comparisons used Tukey's honestly significant difference test when variances were equal and Tamhane's T2 test when they were unequal. After significant nonparametric omnibus tests, pairwise Mann–Whitney *U* tests with Bonferroni correction were applied. Categorical variables were compared using the chi‐square test or Fisher–Freeman–Halton test, as appropriate. Short‐term survival was evaluated by Kaplan–Meier analysis with between‐group differences assessed by the log‐rank (Mantel‐Cox) test. To address residual imbalance in illness‐severity variables within the propensity score–matched cohort, an additional multivariable Cox proportional hazards regression model was constructed for 28‐day mortality. The model included sodium group, Charlson Comorbidity Index (CCI), and Glasgow Coma Scale (GCS). Normonatremia was used as the reference category for sodium‐group comparisons. Hazard ratios (HRs) were reported with corresponding 95% confidence intervals (CIs). Because of the small number of DDEH patients and mortality events, this model was intentionally restricted to a limited number of clinically relevant covariates and was interpreted as an exploratory sensitivity analysis rather than a definitive causal model. All tests were two‐sided, and *p* < 0.05 was considered statistically significant.

## 3 Results

### 3.1 Study Population and Cohort Derivation

During the five‐year study period, 768,677 ED encounters were identified. After application of the prespecified eligibility criteria, including exclusion of pediatric encounters and records without a simultaneous serum/plasma glucose measurement required for sodium correction, the final analytic cohort comprised 227,027 unique adult patients (Figure 1). Within this cohort, 186,194 patients (82.01%) were normonatremic, 38,074 (16.77%) were hyponatremic, and 2759 (1.22%) were hypernatremic.

KDE applied to the continuous Na_corr_ distribution identified a distinct local minimum at 110 mmol/L, which was operationalized as the threshold for DDEH. Using this cutoff, 61 patients met criteria for DDEH, corresponding to a prevalence of 0.027% in the full cohort. Bandwidth sensitivity analyses demonstrated a consistent lower‐tail separation within the corrected sodium distribution. The identified lower‐tail antimodes were 114.67, 114.59, 114.39, and 114.11 mmol/L when bandwidth multipliers of 1.00, 1.25, 1.50, and 2.00 were applied, respectively. These findings indicated that the lower‐tail structure of the sodium distribution was largely preserved across a broad range of smoothing parameters. The 110 mmol/L boundary was retained as a conservative operational definition to identify the most extreme segment of the hyponatremia distribution. Two independent 1:1 PSM procedures were then performed with the DDEH group as the reference, yielding a matched sample of 183 patients (61 per group) across three strata: DDEH (Na_corr_ ≤ 110 mmol/L), nonextreme hyponatremia (110 mmol/L < Na_corr_ < 135 mmol/L), and normonatremia (135 mmol/L ≤ Na_corr_ ≤ 145 mmol/L). Postmatching balance was acceptable for the matching variables, with standardized mean differences (SMDs) < 0.10 for age and sex. However, residual between‐group differences persisted for selected illness‐severity variables, particularly CCI and neurological status, and were therefore addressed in additional adjusted survival analyses.

### 3.2 Baseline Characteristics, Etiology, and Clinical Presentation

The matched cohort had a median age of 71 years (range, 22–93), and age and sex distributions did not differ across strata (*p* = 0.882 and *p* = 0.808, respectively). Comorbidity burden was greater in the DDEH group than in normonatremic controls, with a higher CCI (median 6 vs. 4; *p* = 0.014). Prespecified etiologic categories were broadly similar between groups, without significant between‐stratum differences.

Several physiological and neurological measures varied across sodium strata. SBP was higher in both hyponatremic groups than in the normonatremic group (138 and 129 vs. 121 mmHg; *p* = 0.001), and heart rate showed a similar pattern (91 and 90 vs. 85 bpm; *p* < 0.001). Body temperature decreased stepwise from normonatremia to DDEH (36.9 vs. 36.8°C vs. 36.6°C; *p* < 0.001). Compared with nonextreme hyponatremia, DDEH was associated with more pronounced neurological impairment, including lower GCS scores (14.3 vs. 14.8; *p* = 0.005), more frequent lethargy (16.4% vs. 1.6%; *p* < 0.001), and more frequent loss of consciousness (52.5% vs. 13.1%; *p* < 0.001). Headache, seizure, and coma did not differ significantly across groups. Baseline characteristics are summarized in Table 1.

**TABLE 1:** Baseline clinical characteristics and patient outcomes across different serum sodium levels.

	Distribution‐defined extreme hyponatremia (Na_corr_ ≤ 110 mmol/L) (*n* = 61)	Nonextreme hyponatremia (110 mmol/L < Na_corr_ < 135 mmol/L) (*n* = 61)	Normonatremia (135 mmol/L ≤ Na_corr_ ≤ 145 mmol/L) (*n* = 61)	Test statistic ($\chi^{2}$)	*p*
Demographic data					
Age, years	72 (34–93)	71 (22–93)	71 (22–93)	0.251	0.882
Sex, female, *n* (%)	38 (62.3)	38 (62.3)	41 (67.2)	0.427	0.808
Hemodynamic parameters					
SBP, mmHg	138 (60–190)^b^	129 (73–230)^b^	121 (85–140)^a^	13.743	**0.001**
DBP, mmHg	75 (40–106)	70 (42–112)	70 (43–91)	4.591	0.101
MAP, mmHg	93 (52–126)^ab^	90 (54–146)^a^	87 (57–104)^a^	7.231	**0.027**
Heart rate, bpm	91 (68–170)^b^	90 (61–140)^b^	85 (50–110)^a^	15.101	**< 0.001**
Body temperature, °C	36.6 (36–37.5)^c^	36.8 (36–38.9)^b^	36.9 (36.7–37.7)^a^	46.368	**< 0.001**
SpO_2_, %	91 (85–99)^a^	93 (81–99)^ab^	94 (90–98)^b^	11.108	**0.004**
Clinical scores					
GCS	14 (3–15)^b^	14 (10–15)^a^	14 (13–15)^a^	10.537	**0.005**
CCI	6 (0–12)^b^	5 (0–13)^ab^	4 (0–11)^a^	8.492	**0.014**
Symptoms					
Headache	4 (6.6)	2 (3.3)	0 (0)	3.939	0.168
Lethargy	10 (16.4)^b^	1 (1.6)^a^	0 (0)^a^	15.268	**< 0.001**
Seizure	3 (4.9)	0 (0)	0 (0)	4.114	0.107
Loss of consciousness	32 (52.5)^b^	8 (13.1)^a^	7 (11.5)^a^	34.413	**< 0.001**
Coma	4 (6.6)	1 (1.6)	0 (0)	4.314	0.129
Etiological factors					
Diuretic‐induced	9 (14.8)	9 (14.8)	N/A	< 0.001	> 0.999
Polydipsia	8 (13.1)	3 (4.9)	N/A	1.599	0.206
Poor dietary intake	8 (13.1)	19 (31.1)	N/A	4.756	0.029
Unknown	20 (32.8)	17 (27.9)	N/A	0.155	0.694
Clinical outcomes					
Hospital LOS, days	8 (1–28)^b^	8 (1–28)^ab^	5 (1–28)^a^	8.175	**0.017**
28‐day mortality, *n* (%)	22 (36.1)	11 (18.0)	4 (6.6)	16.735	**< 0.001**

*Note:* Continuous data are presented as median (min–max), and categorical variables are expressed as frequencies and percentages [n (%)], unless otherwise specified. Different superscript letters within the same row indicate statistically significant differences between groups based on post hoc comparisons (*p* < 0.05). Etiologic comparisons were limited to the two hyponatremic groups (DDEH vs. nonextreme hyponatremia); normonatremia was N/A. Bold values in indicate statistically significant *p*‐values (*p* < 0.05).

Abbreviations: CCI, Charlson Comorbidity Index; DBP, diastolic blood pressure; GCS, Glasgow Coma Scale; LOS, length of stay; MAP, mean arterial pressure; N/A, not applicable; SBP, systolic blood pressure; SpO_2_, peripheral oxygen saturation.

### 3.3 Biochemical and Hematological Profiles

Serum chloride and calculated serum osmolality decreased stepwise across sodium strata (70 vs. 92 vs. 100 mEq/L, *p* < 0.001; and 228 vs. 275 vs. 295 mOsm/kg H_2_O, *p* < 0.001, respectively). Glucose was lowest in the DDEH group (112 [61–249] mg/dL) compared with nonextreme hyponatremia and normonatremia (129 [78–811] and 133 [86–536] mg/dL; *p* = 0.002). Calcium, albumin, and total protein were lower in both hyponatremic groups than in normonatremic controls, whereas values were similar between the two hyponatremic strata (all *p* < 0.001).

Renal indices, including urea, BUN, and GFR, did not differ significantly across groups (all *p* > 0.20), although creatinine showed a modest between‐group difference and was lowest in DDEH (*p* = 0.031). Among hematological variables, lymphocyte counts were lowest in DDEH (*p* = 0.019), whereas platelet counts were highest (*p* = 0.002). The DDEH group also showed lower MCV and MPV than both comparator groups (both *p* < 0.001). Detailed laboratory findings are presented in Table 2.

**TABLE 2:** Baseline laboratory findings and calculated laboratory parameters across different serum sodium levels.

Variables	Distribution‐defined extreme hyponatremia (Na_corr_ ≤ 110 mmol/L) (*n* = 61)	Nonextreme hyponatremia (110 mmol/L < Na_corr_ < 135 mmol/L) (*n* = 61)	Normonatremia (135 mmol/L ≤ Na_corr_ ≤ 145 mmol/L) (*n* = 61)	Test statistic (*F*, χ^2^)	*p*
Biochemical parameters					
Glucose (mg/dL)	112 (61–249)^b^	129 (78–811)^a^	133 (86–536)^a^	12.981	**0.002**
Corrected sodium (mEq/L)	107 (94–110)^c^	128 (111–133)^b^	139 (135–145)^a^	161.784	**< 0.001**
Potassium (mEq/L)	4.0 ± 1.0	4.2 ± 1.0	4.3 ± 0.7	1.599	0.205
Chloride (mEq/L)	70 (55–90)^c^	92 (59–106)^b^	100 (85–112)^a^	117.810	**< 0.001**
Calcium (mg/dL)	8.2 (5.5–10.5)^b^	8.3 (3.2–10.7)^b^	8.9 (6.9–10.7)^a^	21.462	**< 0.001**
Magnesium (mg/dL)	1.6 (0.8–2.9)^b^	1.7 (1.1–2.6)^ab^	1.8 (0.9–3.6)^a^	12.053	**0.002**
Urea (mg/dL)	40.4 (9.7–397.9)	51.1 (6.7–234.1)	48.5 (14.6–214.1)	2.261	0.323
GFR (mL/min)	63.6 ± 31.6	59.3 ± 35.2	54.9 ± 32.1	1.067	0.346
BUN (mg/dL)	18.8 (4.5–185.9)	23.8 (3.1–109.3)	22.6 (6.8–100)	2.261	0.323
Creatinine (mg/dL)	0.9 (0.2–10.1)^b^	1.0 (0.2–9.0)^ab^	1.1 (0.4–10.7)^a^	6.964	**0.031**
AST (U/L)	23 (7–787)	21 (7–156)	17 (7–860)	2.348	0.309
ALT (U/L)	14 (4–287)	17 (2–194)	17 (4–806)	1.111	0.574
GGT (U/L)	33 (5–1326)	25 (7–1287)	35 (3–732)	1.164	0.559
ALP (U/L)	94 (34–783)	93 (38–1226)	92 (21–1079)	1.979	0.372
Total bilirubin (mg/dL)	0.8 (0.1–10.3)^b^	0.7 (0.1–25.1)^a^	0.7 (0.1–14.2)^ab^	6.003	**0.050**
LDH (U/L)	258 (85–1946)	232 (103–449)	221 (108–3696)	0.324	0.850
Total protein (g/L)	60 (35–84)^b^	63 (37–93)^b^	68 (52–76)^a^	28.206	**< 0.001**
Albumin (g/L)	33 ± 7^b^	34 ± 7^b^	38 ± 5^a^	12.357	**< 0.001**
CRP (mg/L)	37 (0.6–282)	38 (0.7–353)	16 (0.6–340)	4.322	0.115
Calculated laboratory parameters					
Serum osmolality, mOsm/kg H_2_O	228 (208–286)^c^	275 (231–307)^b^	295 (280–331)^a^	136.832	**< 0.001**
Effective osmolality, mOsm/kg H_2_O	220 (195–226)^c^	265 (225–280)^b^	286 (273–301)^a^	160.657	**< 0.001**
Anion gap (mEq/L)	12 ± 7	12 ± 6	13 ± 5	1.378	0.255
Urea/creatinine ratio	40.7 (11.5–382.3)	43.1 (11.5–185.9)	38.5 (9.4–152.2)	2.938	0.230
Hematological parameters					
WBC (×10^9^/L)	12.0 (3.2–27.7)	11.0 (0.6–72.9)	10.4 (0.4–70.0)	2.331	0.312
Neutrophils (×10^9^/L)	10.3 (1.5–24.7)	9.3 (0.3–68)	7.6 (0.01–43.7)	3.870	0.144
Lymphocytes (×10^9^/L)	0.8 (0.1–2.8)^b^	0.9 (0.06–4.3)^ab^	1.1 (0.2–63.1)^a^	7.961	**0.019**
RBC (×10^12^/L)	3.9 ± 0.7	3.6 ± 1.0	3.9 ± 0.9	1.585	0.208
Platelets (×10^9^/L)	306.3 ± 142.3^b^	237.8 ± 105.2^a^	239.6 ± 105.3^a^	6.568	**0.002**
Hemoglobin (g/dL)	10.9 (6–15)	10.6 (3.8–16.7)	11.2 (4.9–16.5)	0.767	0.682
Hematocrit (%)	30.6 ± 5.2	31.6 ± 8.5	33.6 ± 8.6	2.462	0.088
MCV (fL)	78.3 (58.9–97.5)^b^	87.1 (62.1–113.3)^a^	87.7 (52.3–105.5)^a^	36.904	**< 0.001**
MPV (fL)	9.5 ± 1.0^b^	10.2 ± 1.0^a^	10.3 ± 1.0^a^	10.439	**< 0.001**
Plateletcrit (%)	0.2 (0.1–0.7)	0.2 (0–0.6)	0.2 (0–0.5)	4.451	0.108

*Note:* Continuous data are presented as mean ± standard deviation (SD) or median (min–max), as appropriate. Different superscript letters within the same row indicate statistically significant differences between groups based on post hoc comparisons (*p* < 0.05). Bold values in indicate statistically significant *p*‐values (*p* < 0.05).

Abbreviations: ALP, alkaline phosphatase; ALT, alanine aminotransferase; AST, aspartate aminotransferase; BUN, blood urea nitrogen; CRP, C‐reactive protein; GFR, glomerular filtration rate; GGT, gamma‐glutamyl transferase; LDH, lactate dehydrogenase; MCV, mean corpuscular volume; MPV, mean platelet volume; RBC, red blood cell; WBC, white blood cell.

### 3.4 Clinical Outcomes and Survival Analysis

Hospital LOS differed significantly across strata, with longer stays in both hyponatremic groups than in normonatremic controls (median 8 vs. 8 vs. 5 days; *p* = 0.017). Twenty‐eight‐day mortality increased stepwise across the sodium spectrum, reaching 36.1% in DDEH, 18.0% in nonextreme hyponatremia, and 6.6% in normonatremia (*p* < 0.001).

**FIGURE 2:**
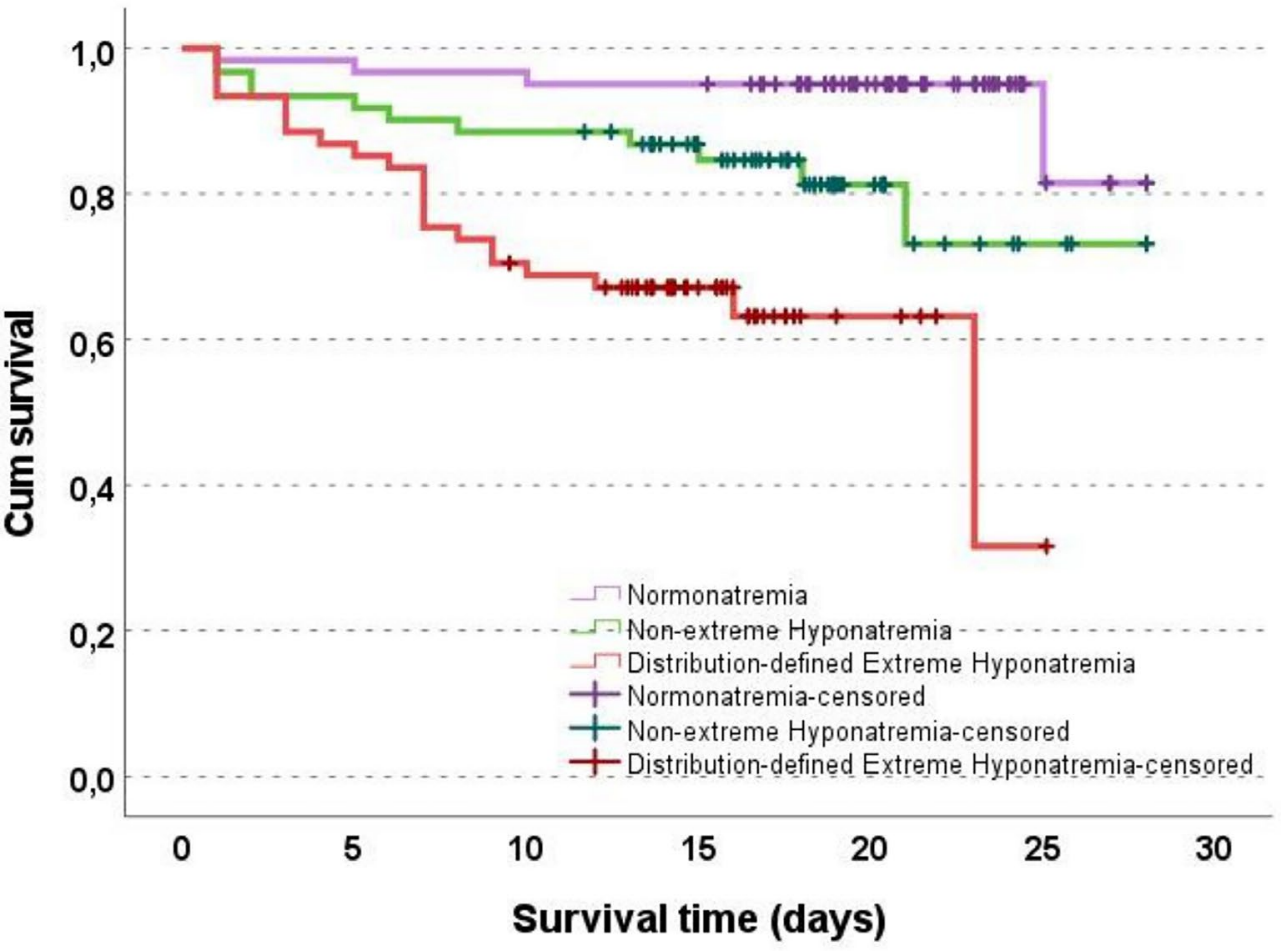
Kaplan–Meier survival curves across sodium‐defined strata. Kaplan–Meier estimates of cumulative survival over 28 days stratified by glucose‐corrected serum sodium categories. Patients were classified as normonatremic, nonextreme hyponatremic, or distribution‐defined extreme hyponatremic based on the KDE‐derived threshold of Na_corr_ ≤ 110 mmol/L. Tick marks indicate censored observations. Survival differences across strata were assessed using log‐rank testing.

Kaplan–Meier analysis demonstrated significant survival differences across groups (Figure 2; log‐rank *χ* ^2^ = 22.512, *p* < 0.001). Estimated mean survival time was 17.6 ± 1.2 days (95% CI, 15.2–20.1) in the DDEH group, 23.8 ± 1.2 days (95% CI, 21.4–26.1) in the nonextreme hyponatremia group, and 26.5 ± 0.7 days (95% CI, 25.0–27.9) in the normonatremic group. Median survival was reached only in DDEH, at 23 days (95% CI, 13.2–32.8), and was not reached in the other two groups during follow‐up. Pairwise log‐rank comparisons were significant for all strata, with the largest separation observed between DDEH and normonatremia (*p* < 0.001). To further explore whether the observed mortality gradient persisted after limited covariate adjustment, a parsimonious multivariable Cox proportional hazards regression model was constructed within the propensity score–matched cohort, adjusting for CCI and GCS. In this model, DDEH remained significantly associated with higher 28‐day mortality compared with normonatremia (HR, 5.46; 95% CI, 1.74–17.17; *p* = 0.004), whereas the association for nonextreme hyponatremia was no longer statistically significant (HR, 2.80; 95% CI, 0.87–9.02; *p* = 0.084). Higher CCI was associated with increased mortality risk (HR, 1.23; 95% CI, 1.09–1.39; *p* < 0.001), whereas higher GCS was associated with lower mortality risk (HR, 0.83; 95% CI, 0.75–0.91; *p* < 0.001) (Table 3).

**TABLE 3:** Multivariable Cox proportional hazards model for 28‐day mortality in the propensity score–matched cohort.

Variables	HR	95% CI	*p*
Sodium group, overall	—	—	0.011
Nonextreme hyponatremia vs. normonatremia	2.804	0.872–9.018	0.084
Distribution‐defined extreme hyponatremia vs. normonatremia	5.460	1.736–17.173	0.004
CCI	1.233	1.092–1.392	< 0.001
GCS	0.825	0.745–0.913	< 0.001

*Note:* The model included sodium group, CCI, and GCS. Normonatremia was used as the reference category for sodium‐group comparisons.

Abbreviations: CCI, Charlson Comorbidity Index; CI, confidence interval; GCS, Glasgow Coma Scale; HR, hazard ratio.

## 4 Discussion

This study identified a distribution‐derived extreme‐tail sodium phenotype that was associated with greater neurological compromise, higher comorbidity burden, and substantially increased short‐term mortality in ED patients. Using a large real‐world cohort and a KDE‐based analytic framework, we identified a local minimum at 110 mmol/L that delineated a rare but clinically vulnerable subgroup enriched for adverse outcomes. Importantly, the present findings should not be interpreted as establishing a definitive biological threshold for mortality risk. Rather, the KDE‐derived boundary identified a cohort‐dependent vulnerability region within the sodium distribution in which severe electrolyte derangement, impaired physiological reserve, and systemic illness burden appeared to converge.

Our cohort‐derived threshold refines existing severity labels by isolating the segment of the sodium spectrum in which risk appears to concentrate most sharply. Current frameworks define profound or severe hyponatremia as < 125 mmol/L [9] or < 120 mmol/L [10], but such pragmatic cutoffs may conceal substantial heterogeneity. In our matched cohort, 28‐day mortality was twofold higher in DDEH than in the nonextreme hyponatremia group (36.1% vs. 18.0%). Compared with previously reported thresholds such as < 116 mmol/L [13] or ≤ 113 mmol/L [12], the KDE‐derived antimode identified a smaller subgroup with disproportionately adverse outcomes. This data‐adaptive approach is therefore best viewed as complementary to consensus thresholds, improving resolution at the extreme tail where risk stratification is most challenging. Additional bandwidth sensitivity analyses showed that the lower‐tail separation of the sodium distribution was preserved across alternative smoothing specifications, with antimodal values clustering around 114 mmol/L. Nevertheless, the 110 mmol/L boundary was retained as a conservative operational definition of the most extreme lower‐tail phenotype rather than as a universal physiological cutoff. Accordingly, the present threshold should be viewed as a cohort‐derived exploratory marker that requires external validation before broader clinical implementation.

The 110 mmol/L threshold also delineated a clinically distinct phenotype. DDEH was associated with more frequent loss of consciousness, lower GCS scores, and more frequent lethargy than the comparison groups. These findings are biologically plausible, given the marked hypo‐osmolality observed in DDEH, which may promote water shifts into the brain and cerebral edema once adaptive volume regulation is exceeded [10, 11, 12]. In practical terms, this boundary may help identify patients who warrant closer neurological observation and carefully titrated osmotic correction in the ED. However, because chronicity of hyponatremia, volume status, measured serum osmolality, urine sodium, urine osmolality, and treatment response data were not consistently available, these findings should be interpreted as clinical associations rather than direct mechanistic evidence.

At the same time, the concentration of neurological compromise at this threshold does not establish whether DDEH is a direct cause of death or a marker of advanced systemic illness. The rise in 28‐day mortality to 36.1% suggests that deeper hyponatremia may cluster with greater illness burden rather than act in isolation. This interpretation is supported by propensity‐matched data showing stepwise increases in mortality with deeper hyponatremia, while cause‐of‐death patterns point to persistent confounding from severe underlying disease, including malignancy and liver dysfunction [26]. In our cohort, DDEH was likewise accompanied by greater comorbidity burden and biochemical features consistent with reduced physiological reserve. Although routinely available renal indices were reported, more granular data on active malignancy, liver dysfunction severity, heart failure status, medication exposure, endocrine causes, and formal SIADH classification were not consistently available, limiting etiologic interpretation. Taken together, these findings support interpreting extreme hyponatremia as a sentinel marker of multisystem vulnerability and reinforce the need for guideline‐concordant osmotic correction alongside parallel stabilization of organ dysfunction in the ED [27]. Consistent with this interpretation, the additional Cox regression model demonstrated that DDEH remained associated with 28‐day mortality after adjustment for CCI and GCS, although this finding should be interpreted cautiously given the limited number of DDEH patients and events. Therefore, the adjusted hazard ratio should be viewed as a hypothesis‐generating estimate rather than a stable or definitive effect size. The persistence of this association suggests that the observed excess mortality was not fully explained by measured comorbidity burden or baseline neurological status. However, because residual confounding from unmeasured illness severity and dynamic in‐hospital variables cannot be excluded, DDEH should be interpreted as a high‐risk vulnerability marker rather than as evidence of an isolated sodium‐driven causal mechanism. This interpretation supports the view that the 110 mmol/L boundary may identify a cluster of multisystem vulnerability rather than an independent sodium‐specific predictor of mortality. Accordingly, DDEH should be regarded as an ED presentation phenotype rather than an etiologic or pathophysiologic subtype of hyponatremia.

Hematologic differences further supported this marker‐based interpretation. Relative lymphopenia in DDEH is compatible with acute stress physiology and neuroendocrine activation, although it remains nonspecific and may also reflect infection, malignancy, or nutritional compromise [28, 29]. Platelet indices suggested a reactive pattern, with higher platelet counts and lower MPV [30, 31]. Most notably, marked microcytosis argues against a purely acute osmotic phenomenon and instead suggests enrichment for chronic comorbidity and diminished hematologic reserve, plausibly related to iron deficiency, malnutrition, or chronic inflammatory states [32, 33]. Although these findings are not specific, they reinforce the impression that DDEH clusters with broader systemic vulnerability rather than representing an isolated electrolyte disturbance.

Clinically, the 110 mmol/L threshold may serve as a pragmatic early warning signal in the ED. Sodium values at or below this boundary may identify patients at high risk of rapid deterioration, thereby supporting earlier escalation of monitoring and more individualized correction planning. At this extreme tail, emergency physicians must balance the immediate risk of cerebral edema from ongoing hypo‐osmolality against iatrogenic harm from overly rapid correction, including ODS [34, 35, 36, 37]. Accordingly, patients meeting this criterion may merit earlier specialist involvement and higher‐acuity observation when clinically indicated, without implying that sodium concentration alone should determine disposition.

Several limitations should be acknowledged. First, the retrospective single‐center design may limit generalizability and introduce selection bias. Second, despite propensity score matching and additional parsimonious Cox regression adjustment, residual confounding related to underlying illness severity, active malignancy, liver dysfunction, heart failure, medication exposure, and incompletely captured etiologic contributors cannot be excluded. The original propensity score model was intentionally restricted to age and sex to preserve stability and retention within the very small DDEH subgroup; however, residual imbalance in CCI and GCS remained and was addressed through adjusted survival modeling. Third, because only 61 patients met the DDEH definition and the number of mortality events was limited, the adjusted Cox estimates had relatively wide confidence intervals and should be interpreted as exploratory rather than definitive causal estimates. This small subgroup size also precluded meaningful subgroup analyses or reliable estimation of effect heterogeneity across etiologic or clinical strata. Fourth, dynamic clinical and etiologic variables during hospitalization, including sodium correction trajectories, chronicity of hyponatremia, changes in volume status, urine sodium, urine osmolality, measured serum osmolality, treatment responses, and evolving organ dysfunction, were not captured. Fifth, all‐cause mortality limits attribution to specific osmotic complications. Finally, KDE‐derived thresholds are data‐adaptive and may vary across populations, kernel specifications, bandwidth selections, and data distributions. Although bandwidth sensitivity analyses supported preservation of the lower‐tail density structure across alternative smoothing parameters, the 110 mmol/L boundary remains a cohort‐derived conservative operational definition rather than a universally generalizable physiological cutoff. External validation in independent multicenter cohorts is therefore required before broader clinical implementation.

Overall, our findings support a distribution‐derived approach to ED risk stratification. In this cohort, a sodium threshold of 110 mmol/L identified a rare but high‐acuity phenotype associated with greater comorbidity, greater neurological impairment, and higher 28‐day mortality. If validated externally, this threshold may help clinicians recognize the extreme tail of hyponatremia as a clinically meaningful state of physiological vulnerability and guide earlier, risk‐informed management.

## Conflicts of Interest

The authors declare no conflicts of interest.

## Data Availability

The data that support the findings of this study are available on request from the corresponding author. The data are not publicly available due to privacy or ethical restrictions.
